# Competition Between Chemolithotrophic Acetogenesis and Hydrogenotrophic Methanogenesis for Exogenous H_2_/CO_2_ in Anaerobically Digested Sludge: Impact of Temperature

**DOI:** 10.3389/fmicb.2019.02418

**Published:** 2019-10-23

**Authors:** Bo Fu, Xin Jin, Ralf Conrad, Hongbo Liu, He Liu

**Affiliations:** ^1^Jiangsu Key Laboratory of Anaerobic Biotechnology, School of Environmental and Civil Engineering, Jiangnan University, Wuxi, China; ^2^Department of Biogeochemistry, Max Planck Institute for Terrestrial Microbiology, Marburg, Germany; ^3^Jiangsu Collaborative Innovation Center of Technology and Material of Water Treatment, Suzhou, China

**Keywords:** methanogenesis, acetogenesis, carbon isotope, temperature, H_2_/CO_2_ utilization

## Abstract

Anaerobic digestion is a widely applied technology for sewage sludge treatment. Hydrogen and CO_2_ are important degradation products, which serve as substrates for both hydrogenotrophic methanogenesis and chemolithotrophic acetogenesis. In order to understand the competition between these processes for H_2_/CO_2_, sludge samples were incubated under H_2_/CO_2_ headspace at different temperatures, and analyzed with respect to turnover of H_2_, CO_2_, CH_4_ and acetate including their δ^13^C values. At 15°C, ^13^C-depleted acetate (δ^13^C of −41 to −43‰) and transient acetate accumulation were observed under H_2_/CO_2_, and CH_4_ accumulated with δ^13^C values increasing from −53 to −33‰. The copy numbers of the *fhs* gene, which is characteristic for acetogenic bacteria, were at 15°C one order of magnitude higher in the H_2_/CO_2_ incubations than the N_2_ control. At 30°C, however, acetate did not accumulate in the H_2_/CO_2_ incubation and the δ^13^C of CH_4_ was very low (−100 to −77‰). At 50°C, isotopically enriched acetate was transiently formed and subsequently consumed followed by the production of ^13^C-depleted CH_4_. Collectively, the results indicate a high contribution of chemolithotrophic acetogenesis to H_2_/CO_2_ utilization at 15°C and 50°C, while H_2_/CO_2_ was mainly consumed by hydrogenotrophic methanogenesis at 30°C. Fermentative production and methanogenic consumption of acetate were active at 50°C.

## Introduction

Anaerobic digestion has been widely used for stabilization and energy recovery of sewage sludge (Kelessidis and Stasinakis, 2012). Anaerobic digestion of organic matter is achieved in four steps: hydrolysis, fermentation, acetogenesis, and methanogenesis (Adekunle and Okolie, 2015). Acetate and CH_4_ are the respective products of chemolithotrophic acetogensis (4 H_2_ + 2 CO_2_ → CH_3_COOH + 2 H_2_O) and hydrogenotrophic methanogenesis (4 H_2_ + CO_2_ → CH_4_ + 2 H_2_O). Chemolithotrophic acetogenic bacteria normally compete directly with hydrogenotrophic methanogens for H_2_/CO_2_ as substrates (Lopes et al., 2015; Liu et al., 2016). Meanwhile, the emission of CO_2_ and CH_4_ during anaerobic digestion of sewage sludge has received attention because of the greenhouse effect (Niu et al., 2013). The generation of acetate instead of CH_4_ from sewage sludge is a promising technology for waste recycling and reduction of greenhouse gas emission (Agler et al., 2011).

Temperature is one of the key variables in anaerobic sludge digestion and has an important effect on H_2_/CO_2_ utilization (Conrad and Wetter, 1990; Kotsyurbenko et al., 2001; Shanmugam et al., 2014). Studies on rice field soils indicate that acetogenic bacteria can outcompete methanogens for H_2_ at low temperature (Conrad et al., 1989; Liu and Conrad, 2011). Thermophilic anaerobic digestion processes offer kinetic advantages when compared with mesophilic conditions. Compared to 35°C, rates of methanogenesis increase at 55°C, but the methanogenic pathway also changes by replacing acetoclastic methanogesis with syntrophic acetate oxidation coupled to hydrogenotrophic methanogenesis (Zábranská et al., 2000; Hao et al., 2011; Ho et al., 2013). Their respective contribution to the overall anaerobic degradation of organic matter in sewage sludge may be different due to different temperatures. Some studies reported the competition between acetogenic bacteria and methanogens in lake sediments and rice field soils (Chin and Conrad, 2010; Liu and Conrad, 2011; Olivier, 2016), however, the effect of temperature on the contribution of acetogenesis and methanogenesis to chemolithotrophic H_2_/CO_2_ utilization in anaerobic digested sludge is not well understood.

However, the differentiation of chemolithotrophic acetogenesis and hydrogenotrophic methanogenesis in H_2_/CO_2_ utilization is complex. Acetate is not only produced by chemolithotrophic acetogenesis but also by fermentation and heterotrophic acetogenesis. Methane is the end product of both acetoclastic methanogenesis and hydrogenotrophic methanogenesis. Isotope technique is a reasonable approach, since studies have shown that the stable carbon isotope fractionation of chemolithotrophic acetogenesis (−38 to −68‰) and hydrogenotrophic methanogenesis (−21 to −71‰) is strong (Galand et al., 2010; Blaser et al., 2013; Gehring et al., 2015; Ji et al., 2018), which imprints a signature on the stable carbon isotope composition (^13^C/^12^C) of acetate and CH_4_.

In this study, we aimed to specify the competition between chemolithotrophic acetogenesis and hydrogenotrophic methanogenesis for H_2_/CO_2_ in anaerobic digested sludge. Incubation under H_2_/CO_2_ at different temperatures served for determining the potential of the chemolithotrophic acetogenesis and hydrogenotrophic methanogenesis. Incubation in the presence of bromoethanesulfonate (BES) was used to inhibit methanogenesis.

## Materials and Methods

### Sewage Sludge Incubation

Sewage sludge was obtained from secondary settling tank sludge of Wuxi Shuofang sewage treatment plant. The physicochemical characteristics of sewage sludge were: pH (7.65); dry weight (DW; 14.3%); volatile substances (72g/L); water content (85.6%); total N (15.8 mg g^–1^ DW); and total phosphorus (17.0 mg g^–1^ DW). Sludge slurries were prepared in 26-mL pressure tubes by mixing 3.9 g sewage sludge and 6.1 mL of anoxic sterile water. The tubes were closed with black rubber stoppers, flushed with N_2_, pressurized to 0.5 bar overpressure, and then pre-incubated at 25°C for about 5 days to deplete alternative electron acceptors and initiate methanogenesis. After pre-incubation, three treatments were all incubated under 15°C, 30°C, 50°C: (1) control, the sludge slurry was incubated under N_2_ headspace; (2) H_2_/CO_2_ treatment, the sludge slurry was incubated under H_2_/CO_2_ (80/20, v/v) headspace to stimulate both chemolithotrophic acetogenesis and hydrogenotrophic methanogenesis; and (3) H_2_/CO_2_ + BES treatment, the sludge slurry was incubated under H_2_/CO_2_ (80/20, v/v) headspace and methanogenesis was inhibited by 100 mM BES. The headspace pressures of the three treatments were all adjusted to 1.5 bar. The tubes with sewage sludge slurry were prepared in numerous parallels (about 108 tubes), of which triplicates were sacrificed for chemical analyses of liquid samples and molecular analyses. Gas samples were taken from 27 tubes during the incubation at few days’ intervals to measure the concentrations of CH_4_, CO_2_, H_2_ and the δ^13^C values of CH_4_ and CO_2_. The other tubes were opened to retrieve liquid samples for analysis of volatile fatty acids (VFAs) concentration and the δ^13^C of acetate, and were stored frozen at −20°C for later molecular analyses. The δ^13^C of the organic carbon in the sewage sludge was −29.8‰.

### Chemical Analysis

Analytical methods for CH_4_, CO_2_, H_2_ in gas samples and acetate in liquid samples were as described before (Fu et al., 2018). Simply, the partial pressures of CH_4_ and CO_2_ were analyzed by gas chromatography (GC). The partial pressures were converted into molar quantities by using the ideal gas volume formula at different temperatures. The small amount of dissolved CH_4_ was neglected, and the amount of dissolved CO_2_ was calculated from the Henry constants at different temperatures. The concentrations of bicarbonate were calculated from the CO_2_ partial pressures and the pH using the equations listed in Stumm and Morgan (1981). The ^13^C content of CH_4_ and CO_2_ was measured using a Finnigan Gas Chromatography Combustion Isotope Ratio Mass Spectrometry System. Concentrations of acetate and other VFAs were analyzed by high-pressure liquid chromatography (HPLC). An HPLC system (Spectra System P1000, Thermo Fisher Scientific, San Jose, CA, United States; Mistral, Spark, Emmen, Netherlands) equipped with an ion-exclusion column (Aminex HPX-87-H) and a Finnigan LC IsoLink (Thermo Fisher Scientific, Bremen, Germany) was used to measure the δ^13^C values of acetate.

### DNA Extraction and Quantification of Gene Copy

DNA was extracted from the sewage sludge sample using the PowerSoil^®^ DNA Isolation kit. Frozen sewage sludge samples were thawed at 4°C. In order to ensure homogeneity, sludge samples were vortexed prior to DNA extraction. Quality and concentration of the extracted DNA were detected by UV spectrophotometer (NanoDrop ND 2000).

All the oligonucleotide primers were synthesized by Shanghai Bio-Engineering Co., Ltd. (China), and all the qPCR reaction components were purchased from Shanghai Bio-Engineering Co., Ltd. (China). The qPCR was conducted in a Rotor-Gene Q fluorescence quantitative PCR instrument. For all assays, the standard was a sample containing known numbers of DNA copies of the target gene. Standards were continuously diluted and used in each reaction to construct calibration curves. Methanogenic archaea and acetogenic bacteria were quantified by amplification of the *mcrA and fhs* genes, respectively using primers listed in *fhs-f*/*fhs-r* Table 1 (Angel et al., 2011; Xu et al., 2015). The *mcrA* and *fhs* gene qPCR conditions included an initial denaturation at 94°C for 4 min, followed 30 cycles at 94°C for 30s at the specific annealing temperature shown in Table 1. In order to know the relative abundance of acetogenic bacteria, we also used the universal primers 519f/907r to quantify the 16S rRNA gene copies of the domain Bacteria (Table 1) (Imachi et al., 2008).

**TABLE 1:** Oligonucleotide sequences used for quantitative PCR (qPCR) approaches.

**Target**	**Primer (reference)**	**DNA sequence (bp)**	**Annealing temperature (°C)**
*mcrA* gene	mlas-mod (Angel et al., 2011)	5′-GGYGGTGTMGGDTTCACMCARTA-3′	57
	mcrA-rev (Angel et al., 2011)	5′-CGTTCATBGCGTAGTTVGGRTAGT-3′	
*fhs* gene	fhs-f (Xu et al., 2009)	5′-gTWTgggAAAAggYggMgAAgg-3′	55
	fhs-r (Xu et al., 2009)	5′-gTATTgDgTYTTRgCCATACA-3′	
Bacteria	519f (Imachi et al., 2008)	5′-CAGCMGCCGCGGTAANWC-3′	50
	907r (Imachi et al., 2008)	5′-CCGTCAATTCMTTTRAGTTT-3′	

## Results

### H_2_/CO_2_ Utilization at Low Temperature

**FIGURE 1:**
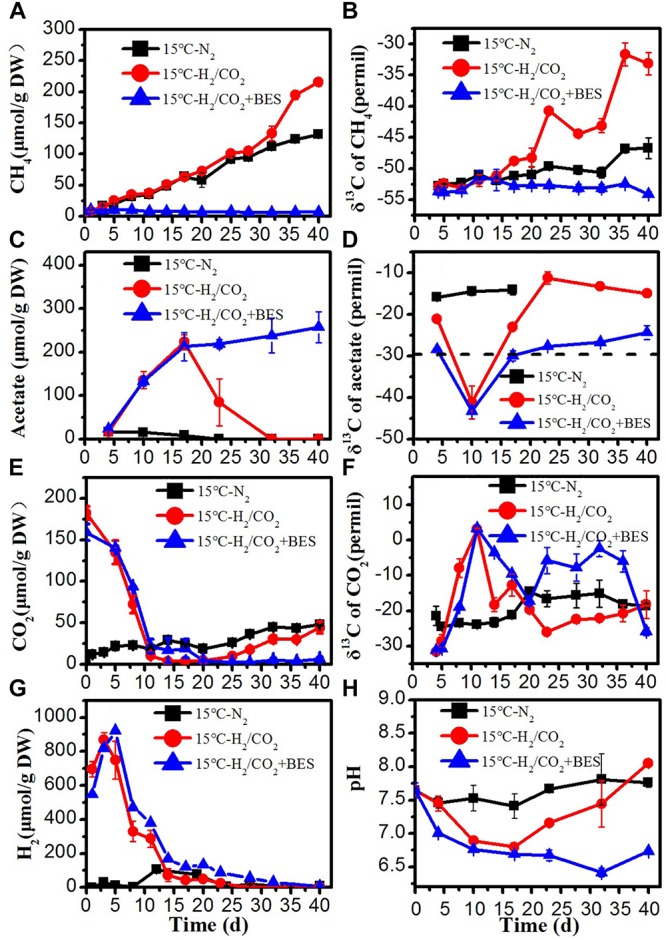
Time course of accumulated **(A)** CH_4_, **(B)** δ^13^C of CH_4_, **(C)** acetate, **(D)** δ^13^C of acetate, **(E)** CO_2_, **(F)** δ^13^C of CO_2_, **(G)** H_2_ concentration, and **(H)** pH during the treatment of sewage sludge at 15°C, BES as an inhibiter of methanogenesis. The δ^13^C of the sludge organic matter (−29.8‰) was represented by dotted line. Mean ± SD, *n* = 3.

**FIGURE 2:**
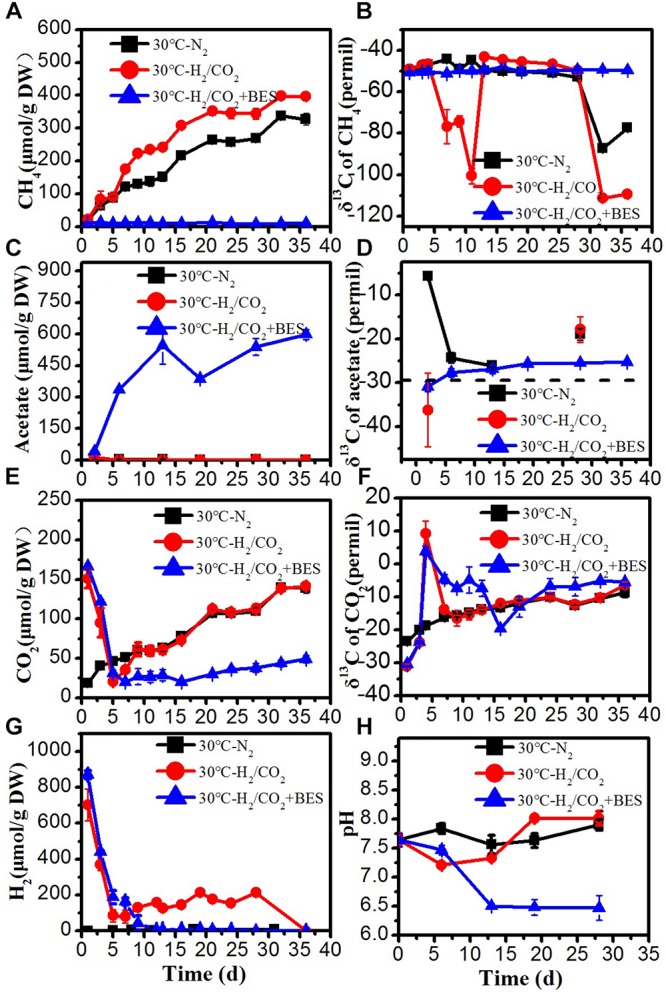
Time course of accumulated **(A)** CH_4_, **(B)** δ^13^C of CH_4_, **(C)** acetate, **(D)** δ^13^C of acetate, **(E)** CO_2_, **(F)** δ^13^C of CO_2_, **(G)** H_2_ concentration, and **(H)** pH during the treatment of sewage sludge at 30°C, BES as an inhibiter of methanogenesis. The δ^13^C of the sludge organic matter (−29.8‰) was represented by dotted line. Mean ± SD, *n* = 3.

**FIGURE 3:**
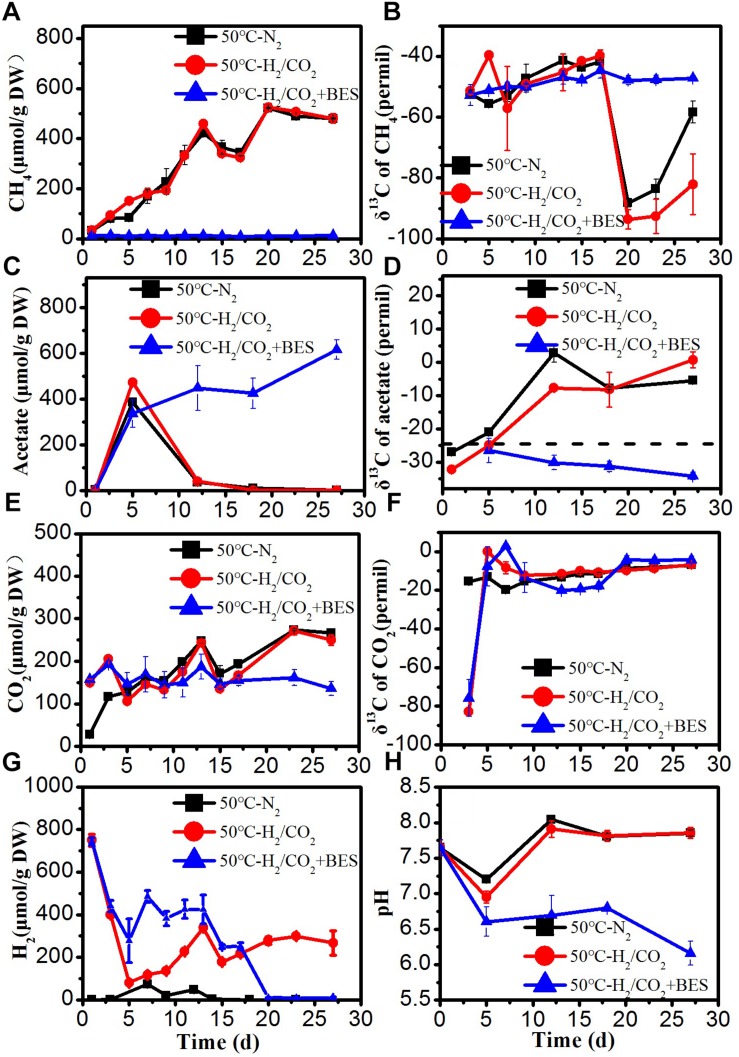
Time course of accumulated **(A)** CH_4_, **(B)** δ^13^C of CH_4_, **(C)** acetate, **(D)** δ^13^C of acetate, **(E)** CO_2_, **(F)** δ^13^C of CO_2_, **(G)** H_2_ concentration, and **(H)** pH during the treatment of sewage sludge at 50°C, BES as an inhibiter of methanogenesis. The δ^13^C of the sludge organic matter (−29.8‰) was represented by dotted line. Mean ± SD, *n* = 3.

The time courses of accumulation of CH_4_, CO_2_, acetate and H_2_, as well as the temporal change of δ^13^C values of CH_4_, acetate, and CO_2_ of the treatments control, H_2_/CO_2_, and H_2_/CO_2_ + BES are shown in Figures 1–3 for the incubation temperatures 15, 30, and 50°C, respectively. At 15°C CH_4_ concentrations increased with time in the control and H_2_/CO_2_ treatments but not in the presence of BES, which inhibited CH_4_ production completely (Figure 1A). At the same time, CO_2_ (Figure 1E) and H_2_ (Figure 1G) concentrations decreased in the H_2_/CO_2_ treatments both in the presence and absence of BES. Later on, CO_2_ slightly increased in the absence of BES presumably because of the conversion of acetate to CO_2_ and CH_4_ (Figure 1E). In the N_2_ incubations H_2_ transiently accumulated to 104 μmol/gDW and then decreased to very low concentration at low temperature (Figure 1G).

The two major products of consumption of H_2_ and CO_2_ were CH_4_ (Figure 1A) and acetate (Figure 1B). In the H_2_/CO_2_ incubations, acetate concentrations accumulated to a maximum on day 17, and then gradually decreased to nearly zero with time (Figure 1B). Acetate was then presumably converted to CH_4_, which was inhibited in the BES-treated samples (Figure 1A). There was almost no acetate accumulation in the N_2_ controls (Figure 1C). Formate, propionate, and butyrate concentrations were always lower than 14, 21, and 23 μmol/g DW, respectively (Supplementary Figure S1).

The amounts of consumed H_2_ and produced acetate and CH_4_ are summarized in Table 2. With exogenous H_2_/CO_2_ and the methanogenic inhibitor BES, about 800–916 μmol/g DW of H_2_ were consumed and about 212–258 μmol/g DW acetate were produced, indicating a stoichiometry of 4 to 1 as expected for chemolithotrophic acetogenesis. Without BES, the transiently accumulated acetate was finally converted to less than 215 μmol/g DW CH_4_, taking into account that CH_4_ was also produced from the sewage sludge without exogenous H_2_/CO_2_.

**TABLE 2:** Accumulated (positive μmol/g DW) or consumed (negative μmol/g DW) metabolites in the different incubations.

	**At time of maximum acetate accumulation**						**At the end**					
		
**Incubation**	**H_2_**	**Acetate**	**Formate**	**Propionate**	**butyrate**	**CH_4_**	**H_2_**	**Acetate**	**Formate**	**Propionate**	**butyrate**	**CH_4_**
15°C, N_2_	–	8	10	8	0	64	–	0	0	0	0	131
15°C, H_2_/CO_2_	−825	223	3	20	0	64	−866	0	0	1	0	216
15°C, H_2_/CO_2_, BES	−801	212	14	21	1	8	−916	258	0	23	6	6
30°C, N_2_	–	2	0	0	0	129	–	0	0	0	0	326
30°C, H_2_/CO_2_	−575	0	23	0	0	222	−700	0	0	0	0	395
30°C, H_2_/CO_2_, BES	−829	335	306	310	71	10	−870	598	0	0	45	10
50°C, N_2_	–	386	0	83	28	84	–	2	0	0	2	479
50°C, H_2_/CO_2_	−669	473	0	86	32	151	−483	2	68	0	2	481
50°C, H_2_/CO_2_, BES	−465	336	11	92	0	11	−734	616	1	82	63	12

The δ^13^C values of acetate under H_2_/CO_2_ treatments showed transiently very low values (<−40‰) on day 10 (Figure 1D). Based on the isotopic signature of acetogenic pure cultures, this ^13^C-depleted acetate was apparently produced from chemolithotrophic acetogenesis (Blaser et al., 2013). These values were much lower than the δ^13^C of sludge organic matter (−29.8‰), indicating that acetate was produced by chemolithotrophic acetogenesis. Later on, δ^13^C values of acetate increased to values >−30‰, especially in the absence of BES, indicating conversion by acetoclastic methanogenesis (Figure 1D). Only little CH_4_ (8–18 μmol/g DW) with a δ^13^C of about −54‰ was observed in in the presence of BES due to the inhibition of methanogenesis. In the absence of BES, the δ^13^C values of CH_4_ under H_2_/CO_2_ increased to about −33‰, but in the N_2_ controls only to about − 47‰ (Figure 1B). In the N_2_ control, the δ^13^C values of CO_2_ accordingly increased from initially −31‰ to about −18.6‰ (Figure 1F). However, in the H_2_/CO_2_ treatments, the δ^13^C values of CO_2_ initially increased to about 0‰, irrespectively of the presence of BES. This increase is consistent with the conversion of CO_2_ to either CH_4_ or acetate. Later on, the δ^13^C values of CO_2_ decreased again, especially in the absence of BES, presumably due to methanogenic consumption of acetate (Figure 1F).

### H_2_/CO_2_ Utilization at Mesophilic Temperature

At 30°C, the time courses of accumulation of CH_4_, CO_2_, acetate and H_2_ are shown in Figure 2. The time courses were similar as at 15°C with the following remarkable exceptions: Methane production rates were larger. Acetate only accumulated in the BES treatment, when CH_4_ production was inhibited by BES (Figure 2B). Similarly, formate, propionate and butyrate accumulated in the H_2_/CO_2_ incubations transiently but only in the presence of BES (Supplementary Figure S2). These observations indicate that any produced VFA was instantaneously consumed and did not accumulate when acetoclastic methanogenesis was operating in the absence of BES. In the N_2_ controls only traces of H_2_ (<7 μmol/g DW) were detected (Figure 2G). The concentrations of H_2_ and CO_2_ both decreased initially in the H_2_/CO_2_ treatments. Although H_2_ and CO_2_ later on gradually increased again but slightly increased H_2_ was completely consumed after Day 28 in the absence of BES (Figure 2E). Initially, H_2_ and CO_2_ was consumed by hydrogenotrophic methanogenesis to produce CH_4_, as indicated by the very low δ^13^C value of CH_4_ (−100.6‰) (Figure 2B). At the end of the incubation, δ^13^C of CH_4_ again decreased to −111.3‰ and δ^13^C of CO_2_ gradually and slightly increased indicating dominance of hydrogenotrophic methanogenesis. The slight increase of H_2_ and CO_2_ concentration in the middle of incubation could be due to the fermentation of organic matter in the sludge, which is consistent with a similar trend and similar values in the N_2_ controls and the decrease of δ^13^C of CO_2_ and the absence of acetate accumulation (Figures 2C,E–G).

The amounts of acetate production (about 500–550 μmol/g DW) were larger than expected from the amounts of H_2_ consumed (about 870 μmol/g DW) and the assumed stoichiometry of 1:4 (Table 2). Accumulation of CH_4_ in the presence of exogenous H_2_/CO_2_ was not much larger (396 μmol/g DW) than in the absence (326 μmol/g DW) (Table 2). Therefore, it is likely that both CH_4_ and acetate were to a large extent produced from the sewage sludge rather than from the exogenous H_2_/CO_2_, which would imply a stoichiometry of 4:1 as characteristic for hydrogenotrophic methanogenesis.

### H_2_/CO_2_ Utilization at Thermophilic Temperature

At 50°C, the rates of CH_4_ production were higher than at 30 and 15°C (Figure 3). The added H_2_ was only slowly consumed when BES was present. The concentrations of H_2_ decreased initially in the H_2_/CO_2_ treatments, but H_2_ later on gradually increased again to the final concentrations of about 280 μmol/g DW, which were higher than at the other temperatures (Figure 3G). The added CO_2_ was also hardly consumed at 50°C, and in the N_2_ control CO_2_ eventually increased to a similar concentration (Figure 3E). The detected H_2_ concentrations in the N_2_ controls were generally lower than 73 μmol/gDW (Figure 3G). Acetate, however, was transiently produced in all the treatments including the N_2_ control, but was later on consumed again except when CH_4_ production was inhibited by BES (Figure 3C). Accumulated formate of 173–206 μmol/g was finally consumed to very low concentration except in the H_2_/CO_2_ treatments where finally about 68 μmol/g DW formate remained (Supplementary Figure S3). In the N_2_ controls and the H_2_/CO_2_ treatments, propionate and butyrate were transiently accumulated to about 87 and 32 μmol/g DW and subsequently consumed (Supplementary Figure S3). Propionate and butyrate concentrations reached 82 and 63 μmol/g DW in the BES treatments (Supplementary Figure S3). The δ^13^C of acetate substantially increased to about − 7‰ due to the consumption, except in the presence of BES (Figure 3D). The δ^13^C of CO_2_ initially increased and then stayed relatively constant at about −15 to −5‰ (Figure 3F), and that of CH_4_ was about −50‰, but decreased significantly at the end of incubation, except in the presence of BES (Figure 3B).

The produced amounts of both acetate and CH_4_ were much larger than the amounts of exogenous H_2_ consumed, assuming a stoichiometry of 1:4 as characteristic for chemolithotrophic acetogenesis and hydrogenotrophic methanogenesis (Table 2). Consequently, it is likely that most of the acetogenic and methanogenic substrates were produced from the anaerobic sewage sludge.

### Quantification of Methanogens and Acetogenic Bacteria

**FIGURE 4:**
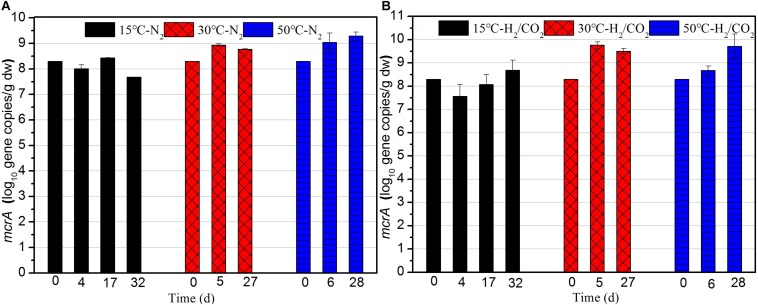
Copy numbers of *mcrA* gene during the **(A)** N_2_ controls and **(B)** H_2_/CO_2_ treatments of sewage sludge at 15, 30, and 50°C. Mean ± SD, *n* = 3.

The copy numbers of the *mcrA* gene, coding for a subunit of the methyl coenzyme M reductase, was measured as equivalent for the number of methanogens in the sewage sludge (Figure 4). In BES treatments *mcrA* was not quantified. The copy numbers of *mcrA* gene at 50 and 30°C were one order of magnitude higher than those of 15°C during the whole incubation (Figure 4A). At 15°C, the final copy numbers of *mcrA* gene under H_2_/CO_2_ were one order of magnitude higher than that of the controls, which indicated H_2_/CO_2_ stimulated the growth of methanogens (Figure 4). The copy numbers of *mcrA* gene at 30°C were always one order of magnitude higher than those of the N_2_ control during the whole incubation. However, the copy numbers of *mcrA* gene in the H_2_/CO_2_ incubation at 50°C were at a similar level than those of the N_2_ controls (Figure 4).

**FIGURE 5:**
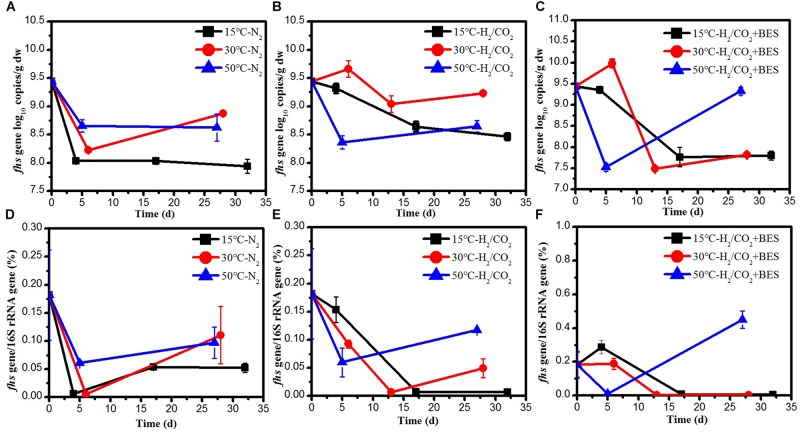
Copy numbers of **(A–C)** *fhs* gene and **(D–F)** *fhs* gene/16S gene during the treatment of sewage sludge at 15, 30, and 50°C, BES as an inhibiter of methanogenesis. Mean ± SD, *n* = 3.

The *fhs* gene, coding for the formyl tetrahydrofolate synthetase, was quantified as equivalent of the number of acetogens, and compared to the number of bacterial 16S rRNA gene copies. At low temperature, the initial copy numbers of *fhs* gene in the H_2_/CO_2_ incubations were one order of magnitude higher than those of the N_2_ control (Figure 5). The copy numbers of *fhs* gene at 30°C showed a same trend as at 15°C and the relative abundance under H_2_/CO_2_ was 19–40 times higher than that of the N_2_ control (Figure 5). At 50°C, addition of H_2_/CO_2_ did not affect the copy numbers and abundance of *fhs* gene (Figure 5).

### Chemolithotrophic Acetogenesis Versus Hydrogenotrophic Methanogenesis Under Elevated H_2_/CO_2_ Concentration at Different Temperature

In order to interpret the competition for H_2_/CO_2_ between acetogens and methanogens at different temperatures, we determined the percentage of methane to the total products (methane + acetate) at the time of maximum acetate accumulation (Table 3). Methanogenesis contributed only marginally (3–4%) in presence of BES due to the inhibition of methanogenesis. However, hydrogenotrophic methanogenesis may has been the exclusive process (98–100%) for H_2_/CO_2_ consumption at 30°C, especially in the treatment with H_2_/CO_2_ (Table 3), which was also indicated by the initial and transient decrease of the δ^13^C of CH_4_ to values of −100.6‰ and final decrease again to −111.3‰ (Figure 2B). By contrast, acetogenesis contributed substantially at 15 and 50°C (Table 3). At 15°C, acetogenesis contributed only in the H_2_/CO_2_ treatment (78%), but at 50°C it also contributed much (82%) without exogenous H_2_/CO_2_ (Table 3). The transient accumulation of acetate at 50°C (especially in the N_2_ control) indicates that at the beginning of the incubation fermentative acetate production (in addition to chemolithotrophic acetogenesis) was faster than the consumption of acetate.

**TABLE 3:** Percentage of methane relative to total products (acetate + methane) formed at the time of maximum acetate accumulation.

**Treatment**	**15°C**	**30°C**	**50°C**
N_2_	89	98	18
H_2_/CO_2_	22	100	24
H_2_/CO_2_ + BES	4	3	3

## Discussion

### The Effect of Temperature on Competition Between Chemolithotrophic Acetogenesis and Hydrogenotrophic Methanogenesis

The competition of acetogens and methanogens for H_2_ is of great importance in many anoxic systems. However, the investigation of the competition between them is very complex. As the product of acetogens, acetate is also produced by fermentation and consumed by different metabolic pathways at the same time. Isotope technique is a reasonable approach to study the competition between acetogens and methanogens for H_2_ since chemolithotrophic acetogenesis and hydrogenotrophic methanogenesis result in a distinct ^13^C depletion of acetate and methane, respectively (Conrad, 2005; Ho et al., 2014; Gehring et al., 2016). Unfortunately, a complication arises from the fact that acetate concentrations in the anoxic environment are often too low for detection and isotopic analysis. Stimulation of chemolithotrophic acetogenesis and hydrogenotrophic methanogenesis by addition of H_2_ apparently allowed determination of reasonable ^13^C values of acetate and methane. Although the experiment set-up of exogenous H_2_ addition may not represent *in situ* condition, it still provides a maximum of further insight into the potential competition between acetogens and methanogens for H_2_.

**FIGURE 6:**
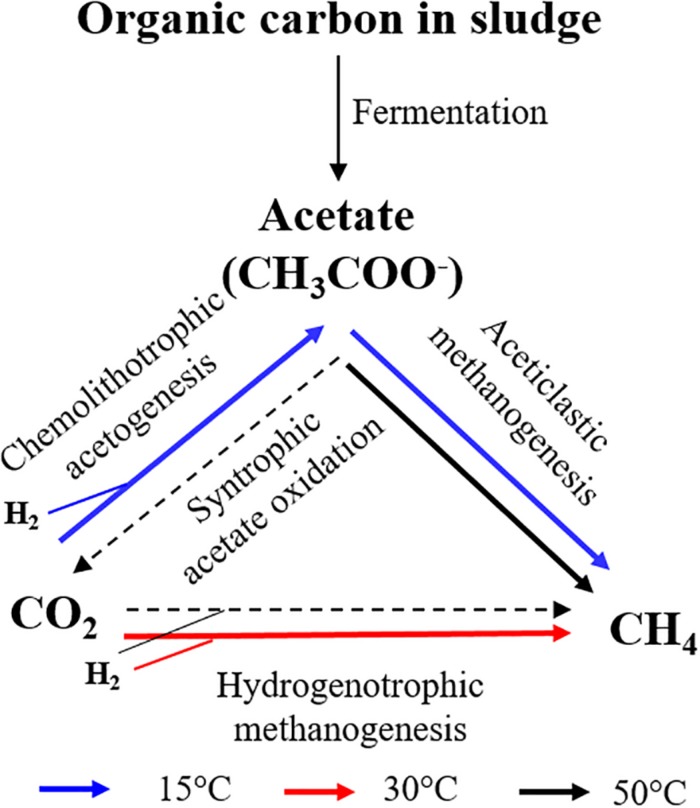
The pathways for consumption of H_2_/CO_2_ during the anaerobic digestion of sewage sludge at 15, 30, and 50°C.

The results of our study showed that the outcome of the competition between chemolithotrophic acetogenesis and hydrogenotrophic methanogenesis strongly depended on the incubation temperature. Collectively, our results suggest the following pathways for consumption of H_2_/CO_2_ (Figure 6). Chemolithotrophic acetogenesis consumed most of the added H_2_/CO_2_ at low temperature (15°C) and high (50°C) temperature. Hydrogenotrophic methanogenesis was the dominant pathway at middle (30°C) temperature. At high temperature, acetate was not only produced from H_2_/CO_2_ but also greatly from organic matter. Subsequently, the acetate was probably degraded by thermotolerant acetoclastic methanogens. A conversion of acetate to H_2_/CO_2_ (by the reversal of chemolithotrophic acetogenesis) was unlikely due to the relatively high H_2_ concentrations in the 50°C treatment, rendering this reaction thermodynamically endergonic.

At 15°C, the addition of H2/CO2 stimulated the production of acetate with isotopically low value (−41.1 to −43.3‰) indicating the operation of chemolithotrophic acetogenesis (Figure 1D). Furthermore, the decrease in δ^13^C values of acetate was paralleled by an increase of copy numbers of the *fhs* gene (Figure 5). The accumulated acetate was gradually exhausted, accompanied by a significant increase of δ^13^C-enriched CH_4_ and an increase of δ^13^C_acetate_ value (Figures 1B,D). Typically, the acetate-derived CH_4_ shows a smaller fractionation than the CO_2_-derived CH_4_ (Conrad, 2005; Gehring et al., 2015). Hence, the formed acetate from chemolithotrophic acetogenesis was mainly consumed by acetoclastic methanogens to produce CH_4_.

At 30°C, the ratios of methane to the total products in the treatments with H_2_/CO_2_ and the N_2_ controls were almost 100% (Table 3). The methane production under H_2_/CO_2_ was accompanied by very low δ^13^C values (−100.5 to −76.8‰) and increased copy numbers of the *mcrA* gene (Figures 2B, 4). This indicated that elevated H_2_/CO_2_ exclusively stimulated the formation of methane via hydrogenotrophic methanogenesis at mesophilic temperature.

At 50°C, the ratios of methane to the total products in the H_2_/CO_2_ incubations and N_2_ controls were only 24 and 18%, respectively. Hence much of the H_2_/CO_2_ was converted to acetate similarly as at 15°C. However, the stoichiometry of acetate production indicated that an additional part was produced from fermentation of organic matters (Heuer et al., 2010). The copy numbers of the *fhs* gene were similar with those in the N_2_ controls. The acetate was transiently produced and paralleled by an increase in δ^13^C values of acetate due to acetate consumption (Figures 3C,D). The isotopically enriched acetate was eventually and completely consumed, followed by the production of ^13^C-depleted CH_4_, which was produced after day 16 until the end of incubation (Figure 3B). Collectively, these observations can be explained by chemolithotrophic acetogenesis from H_2_/CO_2_, followed by aceticlastic methanogenesis. However, the relatively high and constant H_2_ concentrations during the latter incubation are not easily explained. Perhaps, they were caused by small H_2_ production from aceticlastic methanogens (Kulkarni et al., 2018).

Compared to the N_2_ controls, the presence of exogenous H_2_ significantly affected the percentage of methane relative to the total products formed only at 15°C (Table 3), which indicated that chemolithotrophic acetogenesis was more favored at low than at medium and high temperatures. This has also been shown in our previous study of rice field soils (Liu and Conrad, 2011; Fu et al., 2018). Acetogens have at low temperatures higher growth rates than most methanogens (Kotsyurbenko et al., 2001). Under mesophilic conditions, however, methanogenesis is generally energetically more beneficial than acetogenesis, and also exhibits a higher cell-specific affinity for substrate, resulting in much stronger H_2_/CO_2_ utilization via hydrogenotrophic methanogenesis than via homoacetogenesis (Hoehler et al., 2002; Conrad et al., 2008). At thermophilic temperatures, acetate production from H_2_/CO_2_ was augmented by heterotrophic acetate production.

### Implication for Sludge Digestion Operation

This study illuminates the carbon flow in sludge anaerobic digestion under elevated H_2_/CO_2_ concentrations at different temperatures. This understanding deepens our knowledge of methanogenesis pathways involved in anaerobic digestion of sewage sludge, which are fundamental for improvement or regulation of the anaerobic digestion process. Temperature regulation strategy may be used for sludge digestion operation. Thermophilic digestion facilitates syntrophic acetate oxidization, which helps relieve methanogens from substrate inhibition such as high ammonia and high acetate concentration (Hao et al., 2011; Wang et al., 2015; Westerholm et al., 2019). As such, thermophilic digestion could potentially apply to ammonia-rich wastes such as cattle and pig manure or easily degradable wastes such as food waste for methane production.

Methane has a low monetary value. Therefore, more and more attention has been paid to the promises and challenges of an undefined-mixed-culture process to generate a mixture of carboxylates as intermediate platform chemicals toward generation of complex fuels from wastes (Agler et al., 2011). As useful chemical, acetate can be generated from fermentation and homoacetogenesis during anaerobic digestion. Thermophilic digestion enables high hydrolysis and fermentation efficiencies, which could allow efficient acetate accumulation from organic wastes. Additionally, elevated H_2_/CO_2_ concentrations at low temperatures is beneficial to homoacetogenesis, enabling higher production of acetate than methane. Our previous study has reported a novel system coupling glucose fermentation and homoacetogenesis for elevated acetate production (Nie et al., 2008; Ni et al., 2010). When aiming at higher acetate production from sludge, a two-stage thermophilic-psychrophilic AD process coupling fermentation and homoacetogenesis is an alternative approach, with the first stage operated at high temperature (50–55°C) to enable fast hydrolysis and fermentation, and the second stage at 10–15°C under elevated H_2_/CO_2_ concentration derived from the first stage to enable efficient acetogenesis.

## Data Availability Statement

The datasets generated for this study are available on request to the corresponding author.

## Author Contributions

BF planned, designed, and performed the experiments as well as revised the manuscript. XJ participated in performing the experiments and wrote the manuscript. RC designed the experiments and analyzed the results as well as revised the manuscript. HoL assisted in the performance of experiments and revisions of the final manuscript. HeL conceived and coordinated the study, and revised the final manuscript. All authors read and approved the final version of the manuscript.

## Conflict of Interest

The authors declare that the research was conducted in the absence of any commercial or financial relationships that could be construed as a potential conflict of interest.
