# Supplementary material for: Competition Between Chemolithotrophic Acetogenesis and Hydrogenotrophic Methanogenesis for Exogenous H2/CO2 in Anaerobically Digested Sludge: Impact of Temperature
Source: Front Microbiol. 2019 Oct 23;10:2418. doi: 10.3389/fmicb.2019.02418 (PMC6842956; doi:10.3389/fmicb.2019.02418)
Supplement: Supplementary file 1 [file Data_Sheet_1.DOCX]

[Supplement](javascript:;)al materials


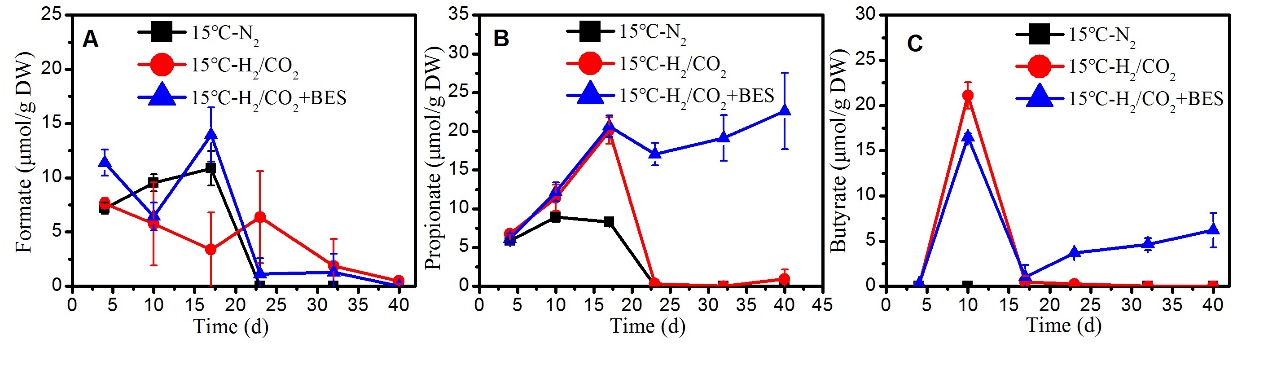
 Figure S1 Time course of accumulated (A) formate, (B) propionate, and (C) butyrate concentration during the treatment of sewage sludge at 15°C, BES as an inhibiter of methanogenesis. Mean ± SD, n = 3.


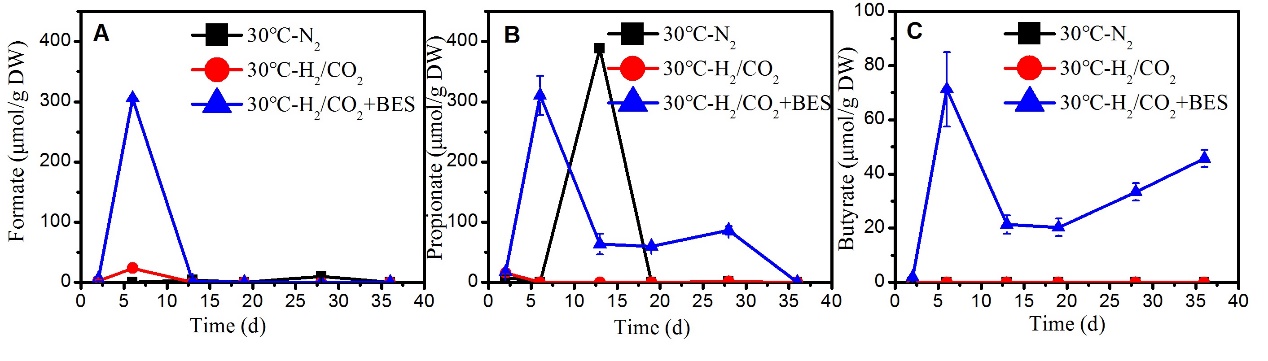


Figure S2 Time course of accumulated (A) formate, (B) propionate, and (C) butyrate concentration during the treatment of sewage sludge at 30°C, BES as an inhibiter of methanogenesis. Mean ± SD, n = 3.


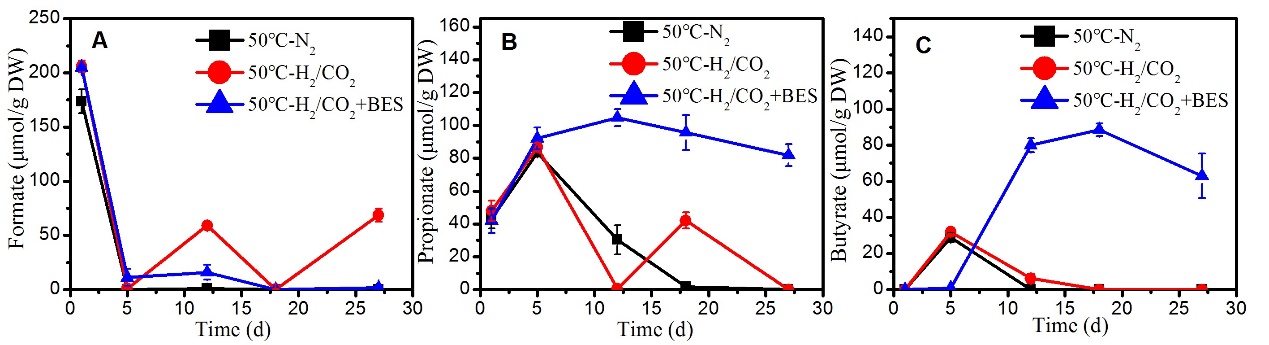


Figure S3 Time course of accumulated (A) formate, (B) propionate, and (C) butyrate concentration during the treatment of sewage sludge at 50°C, BES as an inhibiter of methanogenesis. Mean ± SD, n = 3.
